# An acute postpartum uterine inversion – a near miss case: challenges of management in a resource limited setting – a case report

**DOI:** 10.1097/RC9.0000000000000759

**Published:** 2026-07-28

**Authors:** Tshering Pelden, Namkha Dorji

**Affiliations:** a Faculty of Postgraduate Medicine, Khesar Gyalpo University of Medical Sciences of Bhutan, Thimphu, Bhutan; b Department of Obstetrics and Gynecology, Jigme Dorji Wangchuck National Referral Hospital, Thimphu, Bhutan

**Keywords:** case reports, Huntington procedure, obstetric emergency, postpartum hemorrhage, uterine inversion

## Abstract

**Introduction and importance:**

Acute postpartum uterine inversion is a rare but life-threatening obstetric emergency that can rapidly lead to catastrophic hemorrhage and shock, especially in a resource-limited setting. This case report highlights the importance of prompt diagnosis, timely referral, and multidisciplinary management in achieving favorable maternal outcomes.

**Case presentation:**

A 25-year-old multiparous woman developed acute complete uterine inversion after an uncomplicated vaginal delivery at a lower-level healthcare facility. Initial resuscitative measures were initiated, and attempted manual repositioning was unsuccessful before referral to the national referral hospital. On arrival, the diagnosis was confirmed, and surgical correction was successfully achieved using a combined abdominal traction and vaginal push technique.

**Clinical discussion:**

This case demonstrates the diagnostic and management challenges associated with acute uterine inversion in a resource-limited setting and emphasizes the importance of rapid referral, coordinated multidisciplinary care, and timely surgical intervention.

**Conclusion:**

Acute uterine inversion remains a rare but potentially fatal obstetric event. Appropriate management of the third stage of labor can prevent uterine inversion, and early detection with timely management is essential to improve maternal outcomes.

## Introduction

Uterine inversion is a rare but potentially life-threatening obstetric emergency characterized by the descent or invagination of the uterine fundus into or through the endometrial cavity following childbirth. The reported incidence ranges from 1 in 2000 to 1 in 20 000 deliveries, reflecting variations in obstetric practices, case definitions, and reporting systems^[^1–4^]^. Historically, uterine inversion was associated with high maternal morbidity and mortality because of severe postpartum hemorrhage (PPH) and neurogenic shock, with earlier literature reporting mortality rates as high as 41%^[^5^]^. Although improvements in obstetric care and standardized active management of the third stage of labor (AMTSL) have reduced its incidence globally, cases continue to occur, particularly in resource-limited settings where delayed diagnosis, limited specialist availability, and delayed referral may worsen outcomes^[^6,7^]^.

HIGHLIGHT

- An acute uterine inversion remains a rare but potentially life-threatening obstetric emergency.
- This case demonstrates that correct diagnosis and timely referral from peripheral hospitals can save lives in resource-limited settings.
- Surgical exploration with repositioning of the inverted uterus can be performed successfully.
- Multidisciplinary coordination was critical for optimal maternal outcomes.
- Awareness of the possibility of acute uterine inversion and appropriate management of the third stage of labor are essential to prevent an acute postpartum uterine inversion.

Herein, we report a case of acute complete uterine inversion in a 25-year-old multiparous woman that was managed successfully. This report highlights the diagnostic and management challenges faced by obstetricians working in resource-limited settings and a potential way forward.

## Method

The information presented in this case report was obtained through a retrospective review of the patient’s medical documents and a real-time account from the patient. The report is in line with the SCARE 2025 criteria^[^8^]^.

## Case description

A 25-year-old multiparous woman with previous vaginal deliveries had a normal vaginal delivery at 39^+4^ weeks’ gestation in a lower-level health facility. All low-risk deliveries are managed by nurse-midwives. She had an uneventful antenatal period. She was admitted in labor with 3 cm cervical dilation. Her labor progressed spontaneously, and she delivered a live female baby within 4 hours of admission. Active management of the third stage of labor was performed.

While removing clots, the accoucheur noted a lump inside the vagina with continued vaginal bleeding. In view of PPH, initial uterotonics and resuscitative measures were initiated. Wide-bore IV access was obtained, and an urgent complete blood count showed Hb 7.8 g/dL. An urgent bedside transabdominal ultrasound revealed a postpartum uterus with no clear visualization of the fundus and cervix of the uterus.

Suspecting acute uterine inversion, the general medical doctor on duty consulted the on-call gynecologist at the national referral hospital. As per the advice, manual repositioning of the inverted uterus was attempted, which was not successful. The vagina was then packed with roller gauze, and the patient was referred to the national referral hospital. This referral center has gynecologists on duty around the clock. Upon referral, her blood pressure was 124/77 mmHg, pulse rate was 111 bpm, SpO_2_ was 99% on O_2_ (2 L) via nasal prongs (NP), shock index of 0.9.

Upon reaching the national referral hospital 3 hours and 20 minutes after delivery of the baby, she was severely pale with cold extremities. Blood pressure was 98/75 mmHg, PR-81, SpO_2_ 91% with O_2_ (2 L), shock index of 0.8, with an ongoing transfusion of 1 unit of packed red blood cells (PRBCs). On abdominal examination, the fundus of the uterus was not palpable. Subsequently, upon removal of the packs, the inverted uterus was felt inside the vagina with fresh bleeding. The cervical ring could not be felt. Hemoglobin was 8.6 g/dL. A second attempt was made to manually reposition the uterus in the emergency room, but it was not successful.

**Figure 1.**
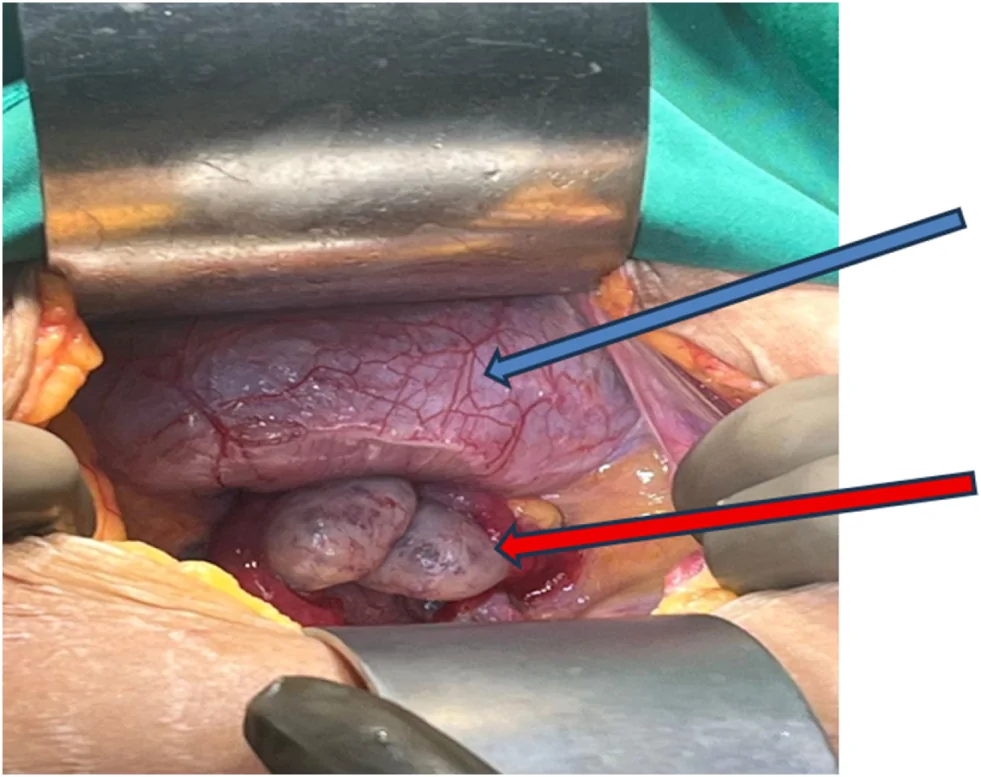
Intraoperative image of an acute postpartum uterine inversion showing the bladder (blue arrow) and bilateral ovaries and fallopian tubes (red arrow) before repositioning.

**Figure 2.**
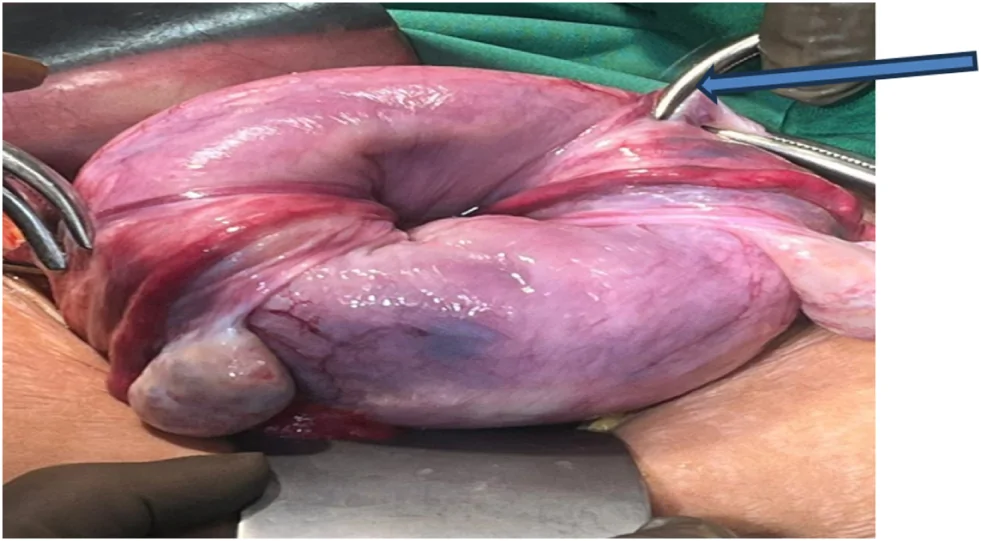
Bilateral round ligaments clamped with Heaney’s forceps (blue arrow) to pull the inverted uterus using Huntington’s procedure while manual pressure is applied vaginally.

**Figure 3.**
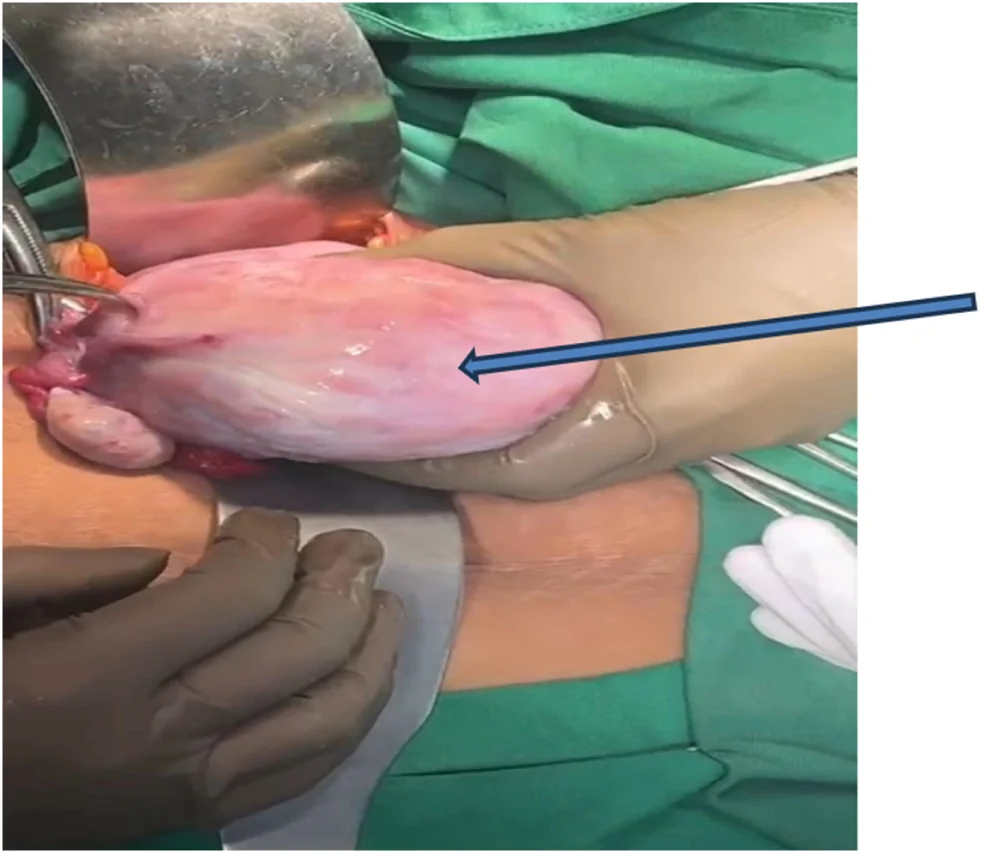
Successfully repositioned uterus (blue arrow) after combining manual vaginal pressure and traction on the round ligaments.

After an unsuccessful attempt, written informed consent was obtained for an emergency laparotomy under general anesthesia to reposition the inverted uterus. The emergency laparotomy confirmed a complete uterine inversion. The uterus was noted to be completely inverted intraoperatively (Fig. 1), with the adnexa closely apposed bilaterally, corresponding to the characteristic “kissing ovaries” (Fig. 1) appearance described in uterine inversion. No active bleeding or intra-abdominal injuries were noted. The round ligaments were clamped bilaterally and pulled to reposition the uterus (Fig. 2). As gentle traction could not reposition the uterus, the first surgeon (N.D.) gently pushed from the vagina using his dominant hand. A combined gentle abdominal pull and gentle vaginal push successfully repositioned the uterus to its anatomical position (Fig. 3).

Following the administration of uterotonics and resuscitative measures, the uterus was well contracted. She was monitored in the intensive care unit overnight and was transferred to the general ward after 24 hours. She was discharged on postoperative day 4.

During routine postnatal care on days 7 and 21, both the mother and the infant were doing well.

## Patient’s perspectives

She expressed her deep gratitude and happiness to the medical team for saving her life and for the care she received at the hospital. She appreciated being fully informed about her case, treatment plans, and future management.

## Discussion

This case illustrates the challenges of recognizing and managing acute postpartum uterine inversion in a resource-limited setting. The nurse-midwife who conducted the delivery recognized the condition immediately. However, it took hours for her to receive definitive treatment at the specialized center. Fortunately, she survived prolonged hypovolemic and neurogenic shock. In addition, active managemnet of third stage of labour (AMTSL) was performed by a nurse midwife in the absence of an obstetrician. This practice is common in delivery centers in Bhutan, as there are a handful of obstetricians stationed in only nine health centers. These difficulties highlight the challenges faced in a resource-constrained setting.

The etiology of uterine inversion is still unclear; however, it is often associated with uncontrolled cord traction or excessive fundal pressure during the third stage of labor. The known risk factors include fetal macrosomia, previous uterine inversion, nulliparity, uterine anomalies, difficult removal of the placenta, placenta accreta, precipitous delivery, short umbilical cord, ligamentous laxity, and the use of uterine relaxants, such as magnesium sulfate^[^1,2^]^.

The diagnosis of acute uterine inversion is primarily clinical, frequently suspected in cases of lower abdominal pain, massive blood loss after delivery, or absence of the uterine fundus during bimanual abdominal palpation, with the greatest diagnostic difficulty arising in first-degree inversion. In fourth-degree inversion, a mass is observed beyond the vaginal introitus. Uterine inversion may be followed by neurogenic shock and subsequently by hemorrhagic shock due to uncontrolled bleeding^[^1,9–12^]^. In a patient with an inconclusive physical examination, an ultrasound examination may confirm the suspected diagnosis by demonstrating a hyperechoic mass in the cervix or vagina, with a hypoechoic cavity in the center^[^13^]^. In our case, the patient was referred within 3 hours of delivery. For the suspected acute uterine inversion with shock (hypovolemic and neurogenic), the patient’s only identifiable risk factor was precipitated labor, which contributes to uterine inversion due to inadequate uterine tone or rapid descent of the fetus^[^6^]^. Excessive or inappropriate cord traction is also a common etiological factor, though this was not documented in this case. The early use of bedside ultrasound at the peripheral hospital was appropriate, but the inability to visualize the uterine fundus underscores the diagnostic challenge in low-resource settings^[^1,9^]^. In Bhutan, ultrasound is usually performed by a technician who lacks anatomical and clinical knowledge. At a remote community hospital in Canada, puerperal uterine inversion was misdiagnosed as placenta accreta^[^9^]^.

Initial attempts to manually reposition the uterus were unsuccessful, which is not uncommon when a cervical constriction ring has already formed. The decision to pack the vagina and refer the patient to a higher center was appropriate, though earlier recognition and referral could have minimized blood loss and the severity of shock. Upon arrival at the referral center, rapid resuscitation, blood transfusion, and timely surgical intervention were decisive in achieving a favorable outcome.

Postpartum uterine inversion is an obstetric emergency that requires immediate recognition, prompt resuscitation, and reversal of the inversion to prevent maternal morbidity and mortality. The goals are to correct hypovolemia and shock, return the uterus to its normal position, and prevent recurrence^[^10^]^. It involves both surgical and nonsurgical methods. Nonsurgical management involves manual replacement (Johnson maneuver), which is the first-line method for acute inversion. The palm and fingers are placed in the vagina, pushing the fundus upward toward the umbilicus through the cervical ring. Sustained pressure, not forceful thrusting, is key to success. Once repositioned, uterotonics (Oxytocin, Methergin) are given to maintain uterine tone and prevent reinversion. Another option is hydrostatic replacement (O’Sullivan method), which is used when manual replacement fails. Warm saline is infused into the vagina using a pressure bag or hydrostatic device, with the vaginal outlet sealed. The hydrostatic pressure gradually reverses the inversion^[^11^]^. When all conservative management fails, surgical methods are applied, which include Huntington procedure, wherein, through laparotomy, the round ligaments or uterine walls are identified and gently pulled upward with atraumatic forceps while the vaginal portion is pushed upward^[^10,11^]^. Another is the Haultain procedure, in which the constricted cervical ring is incised posteriorly to allow repositioning of the uterus, followed by repair of the incision. The choice depends on intraoperative findings and surgeon experience^[^14^]^. In our case, the patient was alert but had low oxygen saturation and a shock index of 0.8 after receiving one PRBC transfusion. Her abdomen was soft, and the uterine fundus was not palpable. After removing vaginal packs, a completely inverted uterus with fresh bleeding was found. A manual repositioning attempt in the emergency room failed. Emergency laparotomy was performed, revealing a complete uterine inversion with “kissing ovaries.” Gentle traction on the round ligaments combined with vaginal upward pressure successfully restored the uterus. Uterotonics were administered; the uterus contracted well; and the abdomen was closed without complications.

Intraoperatively, the uterus was confirmed to be an acute complete (prolapsed) uterine inversion, which often necessitates surgical correction. The surgical technique used – clamping the round ligaments for traction followed by simultaneous push-pull manual repositioning – is consistent with accepted practice in difficult cases, similar to the Huntington or modified Haultain’s approaches described in the literature^[^1,5,10,11,14^]^. The use of uterotonics following repositioning helped restore uterine tone and prevent recurrence.

This case also highlights the critical importance of multidisciplinary coordination, including obstetric surgeons, anesthetists, and nursing teams, which contributed to the successful outcome despite the severity of the condition in a resource-limited setting. Additionally, the insertion of a long-acting contraceptive during recovery constituted appropriate postpartum counseling and preventive care. In our case, the patient agreed to implant insertion.

Overall, this case emphasizes the need for improved training of peripheral health workers to recognize early signs of uterine inversion, perform safe AMTSL, and initiate early referral. Simulation-based training and standardized protocols may reduce delays and improve maternal outcomes in rural settings.

Despite the successful outcome, this case report has several limitations. Firstly, acute puerperal uterine inversion is a clinically important obstetric emergency; its presentation and management are already well described in the literature. Therefore, the novelty of this report lies primarily in highlighting the diagnostic and management challenges encountered in a resource-limited setting rather than introducing a new therapeutic or surgical approach.

Secondly, some aspects of the discussion include broad background information on uterine inversion that may overlap with existing textbook descriptions. The key learning point from this case is the importance of early recognition, timely referral, multidisciplinary coordination, and adaptability of management strategies in lower-level healthcare settings with limited specialist availability.

Thirdly, although conservative methods such as hydrostatic reduction may be considered in selected cases, these options were not feasible at the lower-level health facility due to limited resources, lack of specialized equipment, and delayed presentation with established inversion and ongoing hemorrhage. Immediate surgical intervention at the lower-level health facility was also not possible because specialist obstetric surgical services and anesthesia support were unavailable. These contextual limitations significantly influenced clinical decision-making and referral pathways.

Finally, a graphical abstract or schematic illustration could further improve the educational value and clarity of this report; however, such material was not included in the present report, as high-quality images are available.

## Conclusion

Acute uterine inversion remains a rare but serious obstetric emergency with potentially fatal consequences if not recognized promptly and managed appropriately. This case demonstrates the significant diagnostic challenges present in resource-limited settings and the importance of timely referral and multidisciplinary management at a higher-level center. Early recognition, aggressive resuscitation, and appropriate surgical intervention are key to preventing mortality. Strengthening the skills and confidence of peripheral healthcare providers through ongoing training in AMTSL, emergency obstetric care, and early referral pathways is essential in similar health care settings.

## Data Availability

The data supporting the findings of this case report are included within the article. Additional deidentified data are available from the corresponding author upon reasonable request, subject to patient confidentiality and institutional policies.
